# Less is more

**DOI:** 10.1192/j.eurpsy.2022.1189

**Published:** 2022-09-01

**Authors:** R. Alayón, A. Canalda, R. Albertini, J.M. De Gomar, N. Moro

**Affiliations:** 1 Parc Sanitari Sant Joan de Deu, Unidad De Hospitalización Psiquiátrica Penitenciaria (uhpp-c), Sant Boi de Llobegrat. Barcelona, Spain; 2 Centro penitenciario Wad Ras. Institut Catalá de la Salut, Medicina Familiar Y Comunitaria, Barcelona, Spain

**Keywords:** AV Block, risperidone, paliperidone, EKG

## Abstract

**Introduction:**

Very few research about atrioventricular blocks (AVB) and use of antipsychotic drugs has been made, although it may play an important role in the outcome of any patient affected by psychosis and AVB.

**Objectives:**

To describe a case and review clinical data about AVB progression and neuroleptic treatment.

**Methods:**

We describe a 37 years old inmate male patient who suffered from a first degree AVB and Schizophrenia, being long term treated with neuroleptics (risperidone 9mg/day, switched to paliperidone 9mg/day). Our patient presented very mild symptoms of asthenia and dizziness. An EKG was performed, showing AVB progression to Mobitz Type I^1^. No structural pathology was assessed by ecocardiography. Holter EKG showed also episodes of third degree AV block. Electrophysiology studies were performed showing a supra-hisian AV Block.

**Figure:**
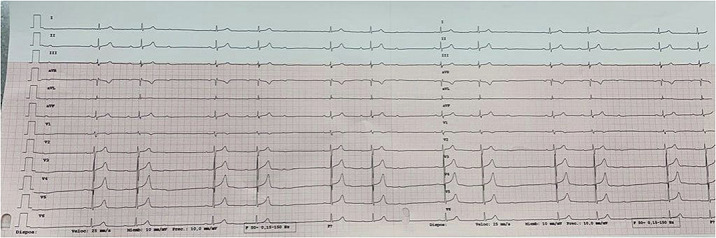

**Results:**

Lower doses of Paliperidone were used (6mg) and maintened until nowadays. Control EKG showed regression to a known first degree AVB.

Being asymptomatic and studies revealing a supra-hisian AVB, no pacemaker was needed.

**Conclusions:**

There is only a few cases described in scientific literature, and very limited data about AVB and neuroleptic drugs, although it is described as possible side effect using risperidone at higher doses. We suggest monitoring EKG to patients affected by AVB, using high doses of neuroleptic drugs. There is no data available about paliperidone metabolites and a possible progression of AVB.

We suggest more studies are needed to better understand and prevent side effects of neuroleptic drugs.

**Disclosure:**

No significant relationships.

